# Serum interleukin-23 levels: relation to depression, anxiety, and disease activity in psoriatic arthritis patients

**DOI:** 10.1007/s10067-022-06300-1

**Published:** 2022-07-21

**Authors:** Samar Abdalhamed Tabra, Salwa Elmorsy abd Elghany, Reham A. Amer, Mohamed H. Fouda, Mohammed Hassan Abu-Zaid

**Affiliations:** 1 Department of Rheumatology and Rehabilitation, Faculty of Medicine, Tanta University, Tanta, Egypt; 2 Department of Neuropsychiatry, Faculty of Medicine, Tanta University, Tanta, Egypt; 3 Department of Clinical Pathology, Faculty of Medicine, Tanta University, Tanta, Egypt

**Keywords:** Anxiety, Depression, Disease activity, HADS score, IL-23, Psoriatic arthritis

## Abstract

**Objectives:**

Assessment of serum levels of IL-23 in PsA patients and its correlation with depression, anxiety, and disease activity.

**Methods:**

Eighty psoriatic arthritis (PsA) patients and eighty healthy volunteers matched for age and gender were included in this observational case–control study. All participants suspected to detailed history, clinical assessment, PsA activity using Disease Activity Index for Psoriatic Arthritis (DAPSA) score, the severity and extent of psoriasis was assessed by the Psoriasis Area and Severity Index (PASI), and ultrasonographic assessments of the entheses were examined according to the Madrid Sonographic Enthesitis Index (MASEI). Depression and anxiety were assessed by Hospital Anxiety and Depression Scale (HADS). Serum IL-23 was measured and correlated with disease activity, depression, and anxiety.

**Results:**

There was no significant difference between patients and controls regarding demographic data. Thirty-six PsA patients (45%) had anxiety and 28 patients (35%) had depression, while in the control group, 16 persons (20%) had anxiety and 12 (15%) had depression, with significant differences between the 2 groups (*p* < 0.0001). There were significant differences in HADS anxiety and depression scores between patients and controls with significant positive correlations between HADS depression, anxiety scores and IL-23, DAPSA, PASI, and MASEI scores (*p* < 0.05). IL-23 was positively correlated with DAPSA, PASI, and HADS scores; we observed that interleukin 23, higher DAPSA, and PASI were independently associated with depression and anxiety.

**Conclusion:**

Serum interleukin-23 levels were elevated in PsA patients and were found to be correlated with depression, anxiety, and disease activity.

**Table:** 

**Key Points** • *Psoriatic arthritis is a multidimensional disorder with psychiatric drawbacks.* • *Interleukin-23 is a proinflammatory cytokines that was correlated with depression and anxiety in PsA patients.* • *Interleukin-23 was correlated with disease activity in PsA.* • *Depression and anxiety were positively correlated with disease activity in PsA.*

## Introduction

Psoriatic arthritis (PsA) affects 0.3 to 1% of the general population and up to 30% of patients with psoriasis [1]. It is a chronic, progressive, inflammatory arthritis associated with cutaneous psoriasis that can potentially affect multiple organ systems. Psoriatic disease is frequently limited to the core clinical domains of skin psoriasis, nail involvement, peripheral inflammatory arthritis, axial spondyloarthritis, enthesitis, and dactylitis, while its numerous systemic consequences and comorbidities are ignored [2]. The psychosocial burden of PsA has been shown to have a negative effect on the quality of life of the patients. In PsA, psychological disorders can both cause and exacerbate disease development [3].

The prevalence of depression in patients with psoriasis ranges from 19.2 to 62% [4–6] and anxiety is reported to be as high as 43% [7]. The presence of joint and skin disease in PsA patients may increase the risk for depression and anxiety [8].

Some existing literature discussed that the proinflammatory cytokines could be elevated in both psoriasis/PsA and depression, which may indicate that the inflammatory process might be involved in the progression of both PsA and depression. Elevated cytokine levels in the central nervous system cause physiologic and biochemical changes that may contribute to the development of depression [9–12].

Interleukin-23 is a proinflammatory cytokine which is involved in the pathophysiology of spondyloarthropathies including psoriatic arthritis and is responsible for many clinical features in PsA [13].

The aim of this study was to assess the serum levels of interleukin-23 in PsA patients and its correlation with depression, anxiety, and disease activity.

## Materials and methods

This is a single-center case–control study.

### Setting

Patients were selected from the outpatient clinic of Rheumatology and Rehabilitation department, Tanta University Hospitals.

### Patients

Eighty patients meeting CASPAR [14] criteria for PsA and 80 healthy volunteers matched for age and gender were included in the study (the healthy control were selected from personnel who work in the hospital and the apparently healthy persons who accompanied the patients). Patients with other dermatological disorders, history of schizophrenia, major depression, other diagnosed psychiatric disorders, and patients on psychotropic drugs were excluded.

Duration of the study: 11 months (from Jan 2021 to Dec 2021). Sample size: regarding previous studies, a total of 80 are required to achieve 80% study power at 95% confidence level with a margin of error equal to 5%. The CONSORT flow diagram was added as supplement 1.

### Ethics approval and consent to participate

This study is in agreement with the ethical guidelines of the Declaration of Helsinki and it follows the ethical standards of Tanta Faculty of Medicine, with the institution’s ethics board approval number 33974/11/20. Informed consent from all patients was obtained in accordance with the local ethical committee. Privacy of all patients’ data was granted as there was a code number for every patient file that included all investigations.

### Clinical assessment

Demographic data and detailed medication history were taken. PsA activity using Disease Activity Index for Psoriatic Arthritis (DAPSA) score [15] was measured using tender and swollen joint counts, patient pain and patient global assessments, and acute phase reactants. Treatment non-response was defined as patients achieving < 5/7 point of the minimal disease activity (MDA) criteria after 3 months of therapy.

The severity and extent of psoriasis was assessed by the Psoriasis Area and Severity Index (PASI) score; a representative area of psoriasis is selected for each body region. The intensity of redness, thickness, and scaling of the psoriasis is assessed as none (0), mild (1), moderate (2), severe (3), or very severe (4) [16].

### Psychiatric assessment

All the participants were subjected to psychiatric evaluation using the Arabic version of mini international neuropsychiatric interview [17]. Patients were evaluated by the Arabic form of Hospital Anxiety and Depression Scale (HADS) (for screening of anxiety and depression) [18] which is a 14-item scale (7 for depression and 7 for anxiety) designed to identify people with anxiety and depression among individuals with medical conditions. Scores for each subscale (HADS-D for depression and HADS-A for anxiety) range from 0 to 21 and can be classified into 3 categories: normal (0–7), borderline abnormal indicating a possible clinical disorder (8–10), and abnormal indicating a probable clinical disorder (11–21).

### Laboratory assessment

- Routine laboratory assessment: erythrocyte sedimentation rate by Westergren method and CRP by ELISA.
- IL-23 was detected in serum by enzyme-linked immunosorbent assay (ELISA): serum was stored at − 70 °C until analysis for IL-23 using a sensitive sandwich ELISA method using the Human IL-23 Immunoassay Quantikine ELISA kit (the minimum detectable dose less than 6.8 pg/mL). The system uses microplates with the walls coated with a monoclonal antibody and an enzyme-linked polyclonal antibody specific for IL-23.

### Radiological assessment

Ultrasonographic assessments of the following entheses were examined bilaterally according to the Madrid Sonographic Enthesitis Index (MASEI) [19]: the inferior pole of the calcaneus, the superior pole of the calcaneus, tibial tuberosity, inferior pole of the patella, superior pole of the patella, olecranon tuberosity. Musculoskeletal ultrasonographic (MSUS) examination was done using (SAMSUNG MEDISON, UGEO) with linear array transducers (7.5–12 MHz). The ultrasound exploration evaluated the following elemental lesions of enthesis at each site: thickness, structure, calcifications, bursae, erosions, and power Doppler signal in bursa or enthesis full tendon (cortical bone profile, intratendon, and paratendon on the enthesis insertion).

Assessment of patients was performed by a rheumatologist experienced in MSUS imaging on the same day of the clinical and laboratory evaluation. Inter-observer reliability: all images were read independently by two physicians blinded to the clinical and laboratory findings.

This study followed the ethical standards of Tanta Faculty of Medicine, with institution’s ethics board approval number 33974; the study was in agreement with the ethical guidelines of the Declaration of Helsinki and informed consent from all patients was obtained in accordance with the local ethical committee. Privacy of all patients’ data was granted as there was a code number for every patient file that included all investigations.

### Statistical analysis

Data were statistically analyzed using SPSS version 20, described in terms of mean ± standard deviation (SD). Student *t*-test for independent samples when variables were normally distributed. For comparing categorical data, chi-square test was performed. Correlation between variables was examined using Pearson’s correlation coefficient. Multiple regression analysis was used to identify variables that may correlate independently with HADS scores. *p* values less than 0.05 were considered statistically significant [20].

## Results

Thirty-three of our PsA patients were oligoarticular type, while 39 were polyarticular, and 8 patients had spondyloarthritis type.

There was no significant difference between patients and controls regarding demographic data. The mean duration of psoriasis was 10.43 ± 2.75 years while the mean duration of PsA was 6.94 ± 4.63 years. Twenty-four patients were on conventional synthetic DMARD (csDMARDs) without biological therapy, while 56 patients were receiving biological therapy (26 were on TNF inhibitors and 30 were on IL-17 inhibitor), 30 patients were receiving combined biologics and csDMARDs, while 26 patients were receiving biologics as monotherapy. Demographic data were mentioned in Table 1.

**Table 1:** Demographic and disease-related characteristics of the PsA patients and controls

	PsA patients (80)	Controls (80)	*p* value
Age (years)	42.15 ± 7.31	39.85 ± 8.75	0.2
Sex (male/female)	44/36	42/38	
BMI	27.65	26.29	
Residence			
Rural	29	24	0.47
Urban	51	56	
Education level			
Elementary	6	4	
High school	12	7	
University	52	59	
Postgraduate	10	10	
Occupation			
Housewife	10	7	
Employment	64	70	0.49
Student	6	3	
Duration of psoriasis (ys)	10.43 ± 2.75	NA	
Duration of psoriatic arthritis (ys)	6.94 ± 4.63	NA	
PsA subtype			
Oligoarticular	33		
Polyarticular	39	NA	
Spondyloarthritis	8		
CRP	8.16 ± 4.85	NA	
ESR (1st hour)	24.5 ± 9.25	NA	
Comorbidities			
Diabetic	6	9	
Hypertensive	4	8	0.91
Cardiac	2	3	
Hyperlipidemia	8	8	
Treatment received		NA	
Methotrexate	54		
Sulfasalazine	10		
Anti-TNF	26		
IL-17 inhibitor	30		

*BMI* body mass index, *CRP* C reactive protein, *ESR* erythrocyte sedimentation rate, *Anti-TNF* anti-tumor necrosis factor, *IL-17* interleukin 17 Significant *p* value if <0.05*

Significant *p* value if <0.05*

According to DAPSA score, 8 patients were in remission, 50 patients had low disease activity, and 20 patients had moderate disease activity, while only 2 patients had severe disease activity. The mean value of PASI score was 22.34 ± 10.91. There was significant difference between patients and controls regarding the serum levels of IL-23 and MASEI score (*p* < 0.0001). Regarding HADS scores, it was found that 36 PsA patients (45%) had anxiety and 28 patients (35%) had depression, while in the control group, 16 persons (20%) had anxiety and 12 (15%) had depression, with significant differences between the 2 groups (*p* < 0.0001). When we compared patients receiving biological therapy with patients who did not receive biologics, we found significant differences in both HADS anxiety and depression scores (*p* < 0.0001) denoting lower levels of anxiety and depression in patients receiving biologics.

At the time of this study, twenty patients had musculoskeletal (MSK) manifestations only, while sixty of them had skin and MSK manifestations, there was significant difference between patients with MSK manifestations only, and who had both skin and MSK manifestations in HADS anxiety and depression scores (*p* = 0.0007, < 0.0001, respectively). Clinical, laboratory, and radiological data were summarized in Table 2.

**Table 2:** Clinical, laboratory, and radiological assessment of the PsA patients and controls

	PsA patients (80)	Controls (80)	*p* value
DAPSA score	11.65 ± 6.82	NA	
Remission (*n*)	8		
Low disease activity (*n*)	50		
Moderate disease activity (*n*)	20		
Severe disease activity (*n*)	2		
PASI score	22.34 ± 10.91	NA	
MASEI score	16.24 ± 11.83	2.56 ± 2.46	< 0.0001*
IL-23 level (pg/mL)	225.19 ± 72.84	118.12 ± 35.64	< 0.0001*
HADS anxiety (in all group participants)	9.87 ± 4.05	8.01 ± 3.87	0.039*
HADS anxiety (in patients on DMARDs only)	13.26 ± 3.12	NA	< 0.0001*
HADS anxiety (in patients on biologics)	6.45 ± 2.23		
HADS anxiety (in patients with MSK manifestations only)	7.76 ± 3.27		0.0007*
HADS anxiety (in patients with skin and MSK manifestations)	10.98 ± 3.64		
Number of anxious persons (%)	36 (45%)	16 (20%)	< 0.0001*
HADS depression (in all group participants)	9.73 ± 2.93	7.43 ± 2.93	< 0.0001*
HADS depression (in patients on cDMARDs only)	13.66 ± 3.75	NA	< 0.0001*
HADS depression (in patients on biologics)	7.19 ± 3.09		
HADS depression (in patients with MSK manifestations only)	6.87 ± 1.57		< 0.0001*
HADS depression (in patients with skin and MSK manifestations)	9.57 ± 2.16		
Number of depressed persons (%)	28 (35%)	12 (15%)	< 0.0001*

*DAPSA* Disease Activity Index for Psoriatic Arthritis, *PASI* Psoriasis Area and Severity Index, *MASEI* Madrid Sonographic Enthesitis Index, *HADS* Hospital Anxiety and Depression Scale, *cDMARDs* conventional disease modifying anti-rheumatic drugs, *MSK* musculoskeletal

Significant *p* value if < 0.05*

**Fig. 1:**
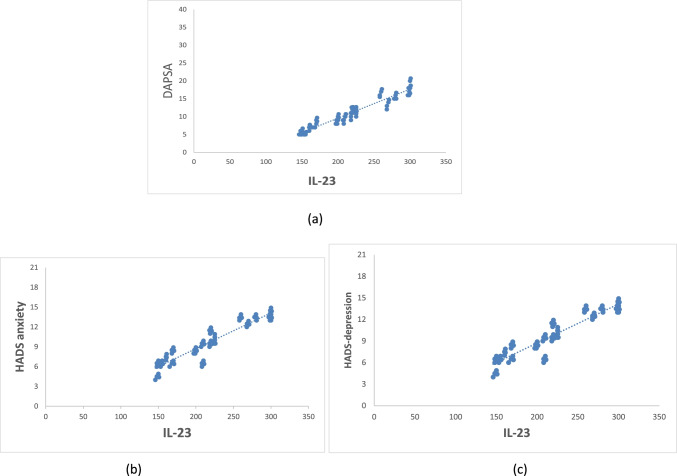
Correlation between serum IL-23 and **a** DAPSA, **b** HADS anxiety, and **c** HADS depression

There was a significant positive correlation between serum IL-23 and DAPSA (*r*: 0. 0.959, *p*: 0.0001), PASI (*r*: 0.765, *p*: 0.0001), MASEI (*r*: 0.545, *p*: 0.001), HADS anxiety (*r*: 0.932, *p*: 0.0001), and depression scores (*r*: 0.934, *p*: 0.0001). Figure 1 demonstrates some of these correlations.

Fifty-eight of our patients were responding to their treatment, while twenty-two of them were non-responders, 20 (90.9%) of the non-responders were anxious, while 16 (27.6%) of treatment responders were anxious (chi-square: 25.8, *p* < 0.0001). Sixteen (72.7%) of the non-responders were depressed, while 12 (20.7%) of treatment responders were depressed (chi-square: 18.9, *p* < 0.0001).

There were significant positive correlations between HADS depression score and IL-23 level, DAPSA, PASI, and MASEI scores (*p*: < 0.0001, < 0.0001, < 0.0001, and 0.0008, respectively). Also, there were significant positive correlations between HADS anxiety score and IL-23 level, DAPSA, PASI, and MASEI scores (*p* < 0.0001, 0.0002, < 0.0001, 0.00003, respectively). Table 3 summarizes the correlations between HADS and other parameters.

**Table 3:** Correlation between HADS and IL-23, DAPSA, PASI, and MASEI score

Value	*r*	*p*
HADS depression		
IL-23 level	0.68	< 0.0001*
DAPSA score	0.55	< 0.0001*
PASI score	0.58	< 0.0001*
MASEI score	0.61	0.0008*
HADS anxiety		
IL-23 level	0.78	< 0.0001*
DAPSA score	0.4	0.0002*
PASI score	0.51	< 0.0001*
MASEI score	0.45	0.00003*

*IL-23* interleukin-23, *DAPSA* Disease Activity Index for Psoriatic Arthritis, *PASI* Psoriasis Area and Severity Index, *MASEI* Madrid Sonographic Enthesitis Index, *HADS* Hospital Anxiety and Depression Scale

Significant *p* value if < 0.05*

With confidence interval (CI = 95%) using the multivariable linear regression analysis to evaluate the factors associated with the presence of depression and anxiety, we observed that interleukin 23, higher DAPSA, and PASI were found to be independently associated with depression and anxiety; these findings were summarized in Table 4.

**Table 4:** Multivariate linear regression analysis for depression and anxiety in PsA patients

	Beta coefficient	*p*
HADS depression		
IL-23	0.513	0.003*
DAPSA	0.292	0.004*
PASI	0.175	0.006*
HADS anxiety		
IL-23	0.486	0.006*
DAPSA	0.465	0.005*
PASI	0.512	0.004*

*IL-23* interleukin-23, *DAPSA* Disease Activity Index for Psoriatic Arthritis, *PASI* Psoriasis Area and Severity Index, *HADS* Hospital Anxiety and Depression Scale

Significant *p* value if < 0.05*

## Discussion

Psoriatic arthritis (PsA) is a chronic progressive inflammatory condition, involving peripheral arthritis and/or spondylitis associated with psoriasis. PsA also affects mental health. This study was undertaken with the intent to assess the serum levels of interleukin-23 in PsA patients and its correlation with depression, anxiety, and disease activity.

Psoriatic patients showed a higher level of anxiety than the control group. According to HADS-A, 45% and 20% of case and control groups had anxiety, respectively. The HADS-D indicated that 35% and 15% of the patients in the case and control groups had depression, respectively.

The results in our study were higher than several previous studies of depression and/or anxiety in PS; this might be explained by the era of COVID-19 and its psychological drawbacks. Geng et al. [21] reported that the prevalence of depression and anxiety is elevated in PsA patients. Among the 114 PsA patients, 31.6% had depression and 13.2% had anxiety. McDonough et al. [8] reported that the prevalence of anxiety was 36.6%, while depression prevalence was 22.2% using HADS. Also, Freire et al. [22] reported a prevalence of anxiety of 29.7% and depression of 17.6%, while Khraishi et al. [23] reported the prevalence of depression and anxiety at 7.9% based on patient self-report.

There are many possible causes of depression and anxiety. Depression and anxiety in PsA patients may be attributed to psoriasis, actively inflamed joint, disability, pain, faulty neurotransmitter systems, stressful life, and fatigue and those, in turn, have a great effect on the quality of life and wellbeing. Also, it has been suggested that depression and anxiety may contribute to the development and progression of PsA [8, 24, 25].

We found that patients with both skin and MSK manifestations had higher HADS anxiety and depression scores. Lada et al. [26] concluded that highlight the increased depressive burden among psoriasis patients with PsA compared to those without PsA.

Regarding response to treatment, we found that anxious and depressed patients were more presented among non-responders to treatment. Kasiem et al. [27] concluded that depression and the combination of a higher BMI with a tender joint count are all associated with MTX non-response.

In our study, higher serum level of IL-23 was correlated with disease activity; Elsawy et al. [28] found that IL-23 is a useful biomarker for identifying joint activity or skin severity. However, Przepiera-Będzak et al. [29] concluded that no correlation of serum IL-6 and IL-23 with VAS, BASDAI, and angiogenic cytokines.

In this study, higher serum level of IL-23, DAPSA score, PASI score, and MASEI score were associated with a higher incidence of both depression and anxiety. This is consistent with McDonough et al. [8] who reported that higher number of active inflamed joint was associated with increased likelihood of depression and anxiety. Also, Milutinovic et al. [30] demonstrated that in axSpA and PsA patients the depression/anxiety symptoms were higher in patients with higher scores in pain, patient global assessment, tender joints count, and increased laboratory parameters of inflammation. Geng et al. [21] reported that the presence of enthesitis in ultrasound and PASI score are associated with depression and anxiety in PsA patients.

It was found that the level of gene expression for IL-17, IL-21, IL-23, and IL-35 was higher in patients with depression [31]. However, Kim et al. [32] do not support a potential involvement of IL-23 and IL-17 axis in major depressive disorder patients. The cytokine hypothesis explains the connection between the immune system and the neuroendocrinal and behavioral alterations that occur in certain forms of depression. Numerous studies demonstrate a 30% elevation of proinflammatory cytokines in patients with depression compared to the healthy population. Inflammatory biomarkers (including interleukin (IL) 1ß, IL-6, tumor necrosis factor-α (TNF-α), C-reactive protein (CRP), adhesion molecules, and prostaglandins) cross the blood–brain barrier and affect the metabolism of neurotransmitters (such as dopamine, serotonin, and glutamate), neuroendocrine function, and even neuroplasticity through decreasing neurotrophic support, neuroprotective function, and neurogenesis in addition to increase in neurotoxicity and neuronal apoptosis [33–36].

IL-23, a member of IL-12 family, is a regulatory factor of inflammatory cytokines, which can be produced by macrophages and dendritic cells. Astrocytes and microglia may also release IL-23. It is an essential cytokine in the differentiation of Th17 lymphocytes. The exact mechanisms of Th17 cell action in the pathology of depression are still unclear. Recent ideas include neuroinflammation, perturbation of dopamine production, IL-17A-mediated glutamate excitotoxicity [37], promotion of neuroprogression, and microglial activation which contribute to neuroprogression [38].

Interleukin 23, higher DAPSA, and PASI were found to be independently associated with depression and anxiety.

Wu et al. [11] reported that older age, female sex, presence of several comorbidities, and psoriatic arthritis were independently associated with depression, while Geng et al. [21] reported that younger age, shorter psoriasis duration, worse pain, and presence of ultrasound enthesitis were associated with depression and severe psoriasis rash is associated with both depression and anxiety in PsA patients. McDonough et al. [8] revealed that being female was associated with an increase in anxiety only, while being unemployed was associated with a higher likelihood of depression and anxiety.

Psoriatic arthritis patients on biological treatment had significant lower score of HADS than patients with conventional DMARDs.

Langley et al. [39] observed a significant correlation between improvement in PASI score and improvement in depression and anxiety score in patients with psoriasis treated with ustekinumab.

Randomized controlled trials on adalimumab, etanercept, and ustekinumab are associated with a statistically significant decrease in depressive symptom scores using different scales in patients with moderate to severe psoriasis [40]. Regarding PsA, Eder et al. concluded that anti-TNFα and IL-12/23 blocker were associated with a significant improvement in depression among PsA patients [41]. In a nationwide cohort study consisting of 980 patients with PsA or psoriasis, Wu et al. [11] assess the effects of biologic therapy on reducing depression and insomnia rates. The prevalence of patients on antidepressants before biologic therapy was 20%. The authors reported that after 2 years of biological therapy there was a reduction of more than 40% in this prevalence rate [11].

Patients treated with biologic DMARDs or other conventional agents have reduced prescription rates for antidepressants and hypnotics, which may suggest that controlling inflammation provides a sufficient improvement in a proportion of patients such that further pharmacotherapy is not warranted [42].

There were some limitations in this study as this is single centered study, it was better if it was a multicenter study, and it was better if the participants were a larger number. Our study is a cross-sectional study; future longitudinal studies are needed to assess the relation between IL-23 and disease activity, depression, and anxiety. Use of a single cytokine at one point of time gives limited information on its role. Also some confounding factors regarding depression and anxiety in this era of COVID-19 pandemic and its effect on psychological status all over the world.

## Conclusion

Serum interleukin-23 levels were elevated in PsA patients and were found to be correlated with depression, anxiety, and disease activity.

## Data Availability

Data will be available when reasonable request.

## References

[1] Gladman D, Antoni C, Mease P, Clegg D, Nash P (2005). Psoriatic arthritis: epidemiology, clinical features, course, and outcome. Ann Rheum Dis.

[2] Alinaghi F, Calov M, Kristensen LE, Gladman DD, Coates LC, Jullien D, Gottlieb AB, Gisondi P, Wu JJ, Thyssen JP, Egeberg A (2019). Prevalence of psoriatic arthritis in patients with psoriasis: a systematic review and meta-analysis of observational and clinical studies. J Am Acad Dermatol.

[3] Husni ME, Merola JF, Davin S (2017). The psychosocial burden of psoriatic arthritis. Semin Arthritis Rheum.

[4] Akay A, Pekcanlar A, Bozdag KE, Altintas L, Karaman A (2002). Assessment of depression in subjects with psoriasis vulgaris and lichen planus. J Eur Acad Dermatol Venereol.

[5] Schmitt JM, Ford DE (2007). Role of depression in quality of life for patients with psoriasis. Dermatology.

[6] Esposito M, Saraceno R, Giunta A, Maccarone M, Chimenti S (2006). An Italian study on psoriasis and depression. Dermatology.

[7] Richards HL, Fortune DG, Griffiths CE, Main CJ (2001). The contribution of perceptions of stigmatisation to disability in patients with psoriasis. J Psychosom Res.

[8] McDonough E, Ayearst R, Eder L, Chandran V, Rosen FC, Thavaneswaran A, Gladman DD (2014). Depression and anxiety in psoriatic disease: prevalence and associated factors. J Rheumatol.

[9] Koo J, Marangell LB, Nakamura M, Armstrong A, Jeon C, Bhutani T, Wu JJ (2017). Depression and suicidality in psoriasis: review of the literature including the cytokine theory of depression. J Eur Acad Dermatol Venereol.

[10] Patel N, Nadkarni A, Cardwell LA, Vera N, Frey C, Patel N, Feldman SR (2017). Psoriasis, depression, and inflammatory overlap: a review. Am J Clin Dermatol.

[11] Wu CY, Chang YT, Juan CK, Shen JL, Lin YP, Shieh JJ, Liu HN, Chen YJ (2016). Depression and insomnia in patients with psoriasis and psoriatic arthritis taking tumor necrosis factor antagonists. Medicine (Baltimore).

[12] De Lorenzis E, Natalello G, Bruno D, Tanti G, Magurano MR, Lucchetti D, Di Mario C, Tolusso B, Peluso G, Gremese E (2021). Psoriatic arthritis and depressive symptoms: does systemic inflammation play a role?. Clin Rheumatol.

[13] Mohanakrishnan R, Beier S, Deodhar A (2022). IL-23 inhibition for the treatment of psoriatic arthritis. Expert Opin Biol Ther.

[14] Taylor W, Gladman D, Helliwell P, Marchesoni A, Mease P, Mielants H (2006). CASPAR Study Group. Classification criteria for psoriatic arthritis: development of new criteria from a large international study. Arthritis Rheum.

[15] Aletaha D, Alasti F, Smolen JS (2017). Disease activity states of the DAPSA, a psoriatic arthritis specific instrument, are valid against functional status and structural progression. Ann Rheum Dis.

[16] Abrouk M, Nakamura M, Zhu TH, Farahnik B, Koo J, Bhutani T (2017). The impact of PASI 75 and PASI 90 on quality of life in moderate to severe psoriasis patients. J Dermatolog Treat.

[17] Sadek A (2000). Mini international neuropsychiatric interview (MINI): the Arabic translation. In: Psychiatry update.

[18] El-Rufaie OE, Absood G (1987). Validity study of the Hospital Anxiety and Depression Scale among a group of Saudi patients. Br J Psychiatry.

[19] de Miguel E, Cobo T, Muñoz-Fernández S, Naredo E, Usón J, Acebes JC, Andréu JL, Martín-Mola E (2009). Validity of enthesis ultrasound assessment in spondyloarthropathy. Ann Rheum Dis.

[20] Kirkpatrick LA, Feeney BC (2013) A simple guide to IBM SPSS statistics for version 20.0 student ed. Belmont, Calif. : Wadsworth, Cengage Learning

[21] Geng Y, Song ZB, Zhang XH, Deng XR, Wang Y, Zhang ZL (2020). Depression and anxiety in patients with psoriatic arthritis: prevalence and associated factors. Beijing Da Xue Xue Bao Yi Xue Ban.

[22] Freire M, Rodriguez J, Moller I, Valcarcel A, Tornero C, Diaz G, Armendáriz Y, Paredes S (2011). Prevalence of symptoms of anxiety and depression in patients with psoriatic arthritis attending rheumatology clinics. Reumatol Clin.

[23] Khraishi M, MacDonald D, Rampakakis E, Vaillancourt J, Sampalis JS (2011). Prevalence of patient-reported comorbidities in early and established psoriatic arthritis cohorts. Clin Rheumatol.

[24] Kotsis K, Voulgari PV, Tsifetaki N, Machado MO, Carvalho AF, Creed F, Drosos AA, Hyphantis T (2012). Anxiety and depressive symptoms and illness perceptions in psoriatic arthritis and associations with physical health-related quality of life. Arthritis Care Res.

[25] Lewinson RT, Vallerand IA, Lowerison MW, Parsons LM, Frolkis AD, Kaplan GG, Bulloch AGM, Swain MG, Patten SB, Barnabe C (2017). Depression is associated with an increased risk of psoriatic arthritis among patients with psoriasis: a population-based study. J Invest Dermatol.

[26] Lada G, Chinoy H, Heal C, Warren RB, Talbot PS, Kleyn CE (2022). Depression and suicidality in patients with psoriasis and the role of psoriatic arthritis: a cross-sectional study in a tertiary setting. J Acad Consult Liaison Psychiatry.

[27] Kasiem FR, Tucker L, Vis M on behalf of CICERO, et al (2022) POS1054 pain and depression are associated with non-response to methotrexate in patients with psoriatic arthritis. Ann Rheum Dis 81:847

[28] Elsawy NA, Helal A, El Shafei M, Mikhael NL (2020). Serum interleukin 23 in psoriatic arthritis patients: relation to disease activity, physical function and health related quality of life. Akt Rheumatol.

[29] Przepiera-Będzak H, Fischer K, Brzosko M (2015). Serum IL-6 and IL-23 levels and their correlation with angiogenic cytokines and disease activity in ankylosing spondylitis, psoriatic arthritis, and SAPHO syndrome. Mediators Inflamm.

[30] Milutinovic S, Veljkovic K, Zlatanovic M, Radunovic G, Damjanov N (2019). Depression/anxiety symptoms in axial spondyloarthritis and psoriatic arthritis patients in Serbia: a pilot study. Rheumatol Int.

[31] Gałecka M, Bliźniewska-Kowalska K, Orzechowska A, Szemraj J, Maes M, Berk M, Su KP, Gałecki P (2021). Inflammatory versus anti-inflammatory profiles in major depressive disorders-the role of IL-17, IL-21, IL-23, IL-35 and Foxp3. J Pers Med.

[32] Kim J-W, Kim Y-K, Hwang J-A, Yoon H-K, Ko Y-H, Han C (2013). Plasma levels of IL-23 and IL-17 before and after antidepressant treatment in patients with major depressive disorder. Psychiatry Investig.

[33] Jokela M, Virtanen M, Batty GD, Kivimäki M (2016). Inflammation and specific symptoms of depression. JAMA Psychiatr.

[34] Valkanova V, Ebmeier KP, Allan CL (2013). CRP, IL-6 and depression: a systematic review and meta-analysis of longitudinal studies. J Affect Disord.

[35] Catena-Dell’Osso M, Bellantuono C, Consoli G, Baroni S, Rotella F, Marazziti D (2011). Inflammatory and neurodegenerative pathways in depression: a new avenue for antidepressant development?. Curr Med Chem.

[36] Capuron L, Miller AH (2011). Immune system to brain signaling: neuropsychopharmacological implications. Pharmacol Ther.

[37] Kostic M, Zivkovic N, Cvetanovic A, Stojanovic I, Colic M (2017). IL-17 signalling in astrocytes promotes glutamate excitotoxicity: indications for the link between inflammatory and neurodegenerative events in multiple sclerosis. Mult Scler Relat Disord.

[38] Swardfager W, Herrmann N, Andreazza AC, Swartz RH, Khan MM, Black SE (2014). Poststroke neuropsychiatric symptoms: relationships with IL-17 and oxidative stress. Biomed Res Int.

[39] Langley RG, Feldman SR, Han C, Schenkel B, Szapary P, Hsu MC, Ortonne JP, Gordon KB, Kimball A (2010). Ustekinumab significantly improves symptoms of anxiety, depression, and skin-related quality of life in patients with moderate-to-severe psoriasis: results from a randomized, double-blind, placebo-controlled phase III trial. J Am Acad Dermatol.

[40] Fleming P, Roubille C, Richer V, Starnino T, McCourt C, McFarlane A (2015). Effect of biologics on depressive symptoms in patients with psoriasis: a systematic review. J Eur Acad Dermatol Venereol.

[41] Eder L, Ogdie A, Zhong Y (2019) Blockage of TNFα and IL-12/23 improves depressive symptoms in patients with psoriatic arthritis – analysis of clinical trial data [abstract]. Arthritis Rheumatol 71(suppl 10)

[42] Brenner P, Citarella A, Wingård L, Sundström A (2020). Use of antidepressants and benzodiazepine-related hypnotics before and after initiation of TNF-α inhibitors or non-biological systemic treatment in patients with rheumatoid arthritis, psoriatic arthritis or ankylosing spondylitis. BMC Rheumatol.

